# Effects of different rotation cropping systems on potato yield, rhizosphere microbial community and soil biochemical properties

**DOI:** 10.3389/fpls.2022.999730

**Published:** 2022-09-29

**Authors:** Junhong Qin, Chunsong Bian, Shaoguang Duan, Wanxing Wang, Guangcun Li, Liping Jin

**Affiliations:** Institute of Vegetables and Flowers, Chinese Academy of Agricultural Sciences/Key Laboratory of Biology and Genetic Improvement of Tuber and Root Crop, Ministry of Agriculture and Rural Affairs, Beijing, China

**Keywords:** potato, continuous cropping, yield, soil biochemical properties, soil microbial communities

## Abstract

Continuous potato cropping systems cause yield reduction, soil-borne disease aggravation, and soil degradation, but crop rotation can alleviate these negative effects. However, there are limited studies on the relationships between microbial community and other soil biochemical properties of continuous potato cropping at both pre-planting and harvest in North China. A 4-year study was conducted to explore the effects of different rotation system on soil biochemical properties, microbial community at pre-planting and harvest, and potato yield, tuber number and black scurf incidence at harvest in 2020 and 2021, which included 4 treatments vis. potato-potato-potato-potato (PC), potato-oat-faba bean-potato (PR), oat-faba bean-potato-oat (O), and faba bean-potato-oat-faba bean (B). The results showed that soil biochemical properties and microbial community among all treatments showed no significant difference at pre-planting after a long cold winter generally. At harvest, PC reduced tuber yield and number and significantly increased black scurf incidence relative to potato rotation systems. PC also reduced soil enzyme activities, the content of soil nutrients, and fungal community diversity, and increased bacterial community diversity compared with the other treatments, insignificantly when compared with PR. Relative abundance of microorganisms related to the degradation of organic residues, soil nitrogen cycling, and disease suppression, such as the genera *Devosia*, *Aeromicrobium*, *Paraphoma*, and *Papiliotrema*, were significantly higher in O or B than in PC and PR, while microorganisms related to disease infection such as the genera *Pseudomonas*, *Colletotrichum*, *Plectosphaerella*, *Fusarium*, and *Verticillium* exhibited increased in PC and PR. Principal Coordinates Analysis (PCoA) showed that there were significant differences in the microbial community structure of PC and PR at harvest compared with that of O and B. Redundancy analysis (RDA) revealed that soil available potassium (AK), acid phosphatase (ACP), available phosphorus (AP), sucrase (SUC) and pH were the dominant factors that significantly affected bacterial and fungal community structure. Partial least squares structural equation model indicated rotation system had significant negative effect on fungal community. It was concluded that growing oat or faba bean after potato can increase soil beneficial microorganisms and maintain the ecosystem healthy, thus reducing the incidence of tuber black scurf and increasing potato yield.

## Introduction

Potato (*Solanum tuberosum* L.) is ranked as the fourth food crop globally after rice, wheat, and corn. In recent years, potato growers in China have had to cultivate potato consecutively due to limited arable land and the adjustment of planting structures in this country. Consequently, potato yield decreased by 27–85% (Qin et al., 2017), soil-borne disease incidence such as black scurf and common scab increased by 25.9–235.7% and 27.1–60.2%, respectively (Larkin et al., 2011), and soil fertility such as available K and P decreased by 38.1–285.6% and 51.2–59.8%, respectively (Wang et al., 2022). In order to alleviate the problems above, the growers used large amounts of fungicides to control soil-borne disease and applied more and more chemical fertilizer to improve potato yield, which may cause potential environment pollution. Crop rotation is an environment friendly practice that could potentially solve the problems mentioned above (Larkin et al., 2021). For example, green manure-potato rotation (Wang et al., 2022), legume-potato rotation (Qin et al., 2017), and potato-canola-wheat rotation (Mohr et al., 2011) can maintain soil fertility and keep the ecosystem healthy, and thus significantly improving potato yield relative to continuous potato cropping.

Soil microorganisms are instrumental in nutrient cycling and transformation (Kuypers et al., 2018) and soil structure development (Helliwell et al., 2014). Under a cucumber continuous cropping system, the balance of the structure of the soil microbial community was broken, with soil microorganisms shifting significantly from bacteria to fungi (Ma et al., 2004). Tan et al. (2022) recently reported that the diversity of soil bacterial and fungal communities increased and decreased in continuous potato cropping, respectively, but Liu et al. (2014) showed that the diversity of soil bacteria decreased with an increase in continuous cropping years, while the diversity of fungal community was mostly unaffected. This was partially congruent with results from She et al. (2017) that bacterial diversity decreases with continuous cropping years in tobacco. However, Potter et al. (2022) found that the diversity of soil bacterial and fungal community did not differ among maize cropping systems. Beneficial microflora such as *Sphingomonas* and *Haliangium* decreased and pathogenic fungi such as *Fusarium* and *Stagonosporopsis* increased with the continuous years of potato cropping (Wang et al., 2022). *Fusarium* can cause potato stem, root, and tuber rot (Yang et al., 2019). Soil chemical properties are closely related with soil microorganisms. For example, in the continuous cropping of strawberry (Huang et al., 2018) and tobacco (She et al., 2017), soil pH tended to decrease over time and was correlated with low bacterial diversity. With potato, however, soil pH increased with time and had positive and negative correlation with bacterial and fungal content, respectively (Wang et al., 2022). By contrast, Xu et al. (Xu et al., 2015) reported a significant decrease in soil pH with the continuous cropping years. Soil enzymes have a strong positive relationship with bacterial abundance (Taylor et al., 2002). In continuous potato cropping, soil urease and alkaline phosphatase activities gradually decreased, and the numbers of soil bacteria and fungi had significant relationships with alkaline phosphatase activity (Qin et al., 2017). Overall, the relationship between soil biochemical properties and microbial community under continuous cropping systems varied with climate conditions, soil texture, field management and crop species.

In Hebei Province, More than 80% of the potato cultivated in the north, which is the main potato production region in North China. In recent years, continuous cropping obstacles caused problems such as yield reduction and soil-borne disease aggravation became more and more serious in North China (Xu et al., 2019). So, it is urgent to explore the key factors caused these problems and select optimum rotation system to alleviate these problems. However, studies on the continuous cropping system are mainly focus on few single factors such as microbial communities or crop yield at harvest. It is still unclear that how the soil microbial communities change at pre-planting after a long cold winter. In this study, a 4-year oat/faba bean/potato rotation trial with different rotation sequences was conducted in North China from 2018 to 2021 to investigate: 1) the potato yield and soil-borne disease incidence in PC (4-year potato continuous cropping) and PR (potato rotation system, viz. potato oat-faba bean-potato); 2) the soil biochemical properties and microbial community composition in different rotation systems at pre-planting and harvest; and 3) to determine the key soil biochemical properties that influenced on soil microbial community composition in potato rotation systems.

## Materials and methods

### Description of the experimental site

The experiment was conducted from 2018 to 2021 at the experimental station (41°25′00″ N, 114°55′57″ E, 1450 m a.s.l.) of the Institute of Vegetables and Flowers of Chinese Academy of Agricultural Sciences in Chabei Administration District, Zhangjiakou City, Hebei Province, China. The region has an average temperature of 2.9°C and an annual precipitation of 381.4 mm. Only one crop per year is cultivated due to the climate features. The soil type was clay loam and its bulk density and field capacity were 1.44 g·cm^-3^ and 24.33%, respectively. D668 (potato genotype), Jican 1(faba bean), and Bayou 18 (fodder oat genotype) were used as crop materials. The potato seeds were produced by our research group, while the seeds of the other two crops were provided by Zhangjiakou Academy of Agricultural Sciences.

### Experimental design and soil samplings

The experiment commenced in 2018 and included four crop sequences: 1) potato-oat-faba bean-potato (PR), 2) oat-faba bean-potato-oat (O), 3) faba bean-potato-oat-faba bean (B), and 4) potato-potato-potato-potato (PC). A randomized block design with three replications was used, and each plot size was 500 m^2^ (50 m ×10 m) to facilitate mechanized management. The row distance and plant spacing of potato were 0.9 m and 0.18 m, respectively. The oat planting density was 4.5×10^6^ plants·ha^-1^ with a row distance of 0.20 m, and the faba bean planting density was 0.33 m (row distance) × 0.12 m (plant spacing). Every year, compound fertilizer (N:P:K=12-19-16) was applied at a rate of 700 kg·ha^-1^ at pre-planting, while nitrogen and potassium were applied during the plant development stage at a rate of 150 kg·ha^-1^ (urea) and 150 kg·ha^-1^ (potassium sulfate), respectively. All plots were fallow before the experiment commenced. Potato and the other two crops were planted in mid- and late-May, respectively, and all crops were harvested in mid-September each year. The investigation began at harvest in 2020 and 2021 for potato yield. At pre-planting and harvest in 2021, soil samples from all crops were collected at five points (“X” sampling method) from each plot and then mixed as one soil sample. Before planting, surface soil (0-20 cm) was collected. At harvest, the whole root of the crop (three plants per point) was completely dug out with a shovel, shaken gently, and then the soil adhering to the root was collected. Samples were passed through a 2-mm sieve and divided into two parts—one was stored at −80°C for microorganism analysis and the other was used for biochemical analysis (Wang et al., 2022).

### Analysis of soil biochemical properties

Soil pH was measured with a glass electrode (HQ440D, HACH, USA) (water:soil = 2.5:1). Soil available phosphorus (AP), available potassium (AK), and available nitrogen (AN) were determined by molybdenum blue colorimetric method, flame photometry method, and the alkaline hydrolysable diffusion method, respectively. Soil organic matter (OM) was determined by potassium dichromate titration method (Bao, 2000). Soil acid phosphatase (ACP), alkaline phosphatase (ALP), sucrase (SUC), and urease (URE) were measured using a soil enzyme activity assay kit (Beijing Solarbio Co., Ltd. Beijing, China)

### Yield evaluation

The potato yield was determined from each plot at harvest in 2020 (O treatment) and 2021(PR treatment). The black scurf incidence of tubers in 2021 was calculated by the number of tubers with symptoms divided by the total number of tubers per plants and then multiplied by 100.

### Soil DNA extraction and illumina sequencing

A PowerSoil DNA Isolation Kit (MoBio Laboratories, Carlsbad, CA, USA) was used to extract soil DNA following the manufacturer’s instructions. Purity and quality of the extracted genomic DNA were checked on 1% agarose gels and with a NanoDrop spectrophotometer (Thermo Scientific). The V3-V4 hypervariable regions of bacterial 16S rRNA gene were amplified with primers 338F (5′-ACTCCTACGGGAGGCAGCAG-3′) and 806R (5′-GGACTACNNGGGTATCTAAT-3′). Fungal ITS genes were amplified with the primers ITS5F (5′-GGAAGTAAAAGTCGTAACAAGG-3′) and ITS1R (5′-GCTGCGTTCTTCATCGATGC-3′). PCR amplification was conducted on a Mastercycler Gradient (Eppendorf, Germany) and the cycling parameters were 94°C for 4 min, followed by 28 cycles (16S rRNA gene) or 34 cycles (ITS gene) at 94°C for 30 s, 55°C for 30 s, and 72°C for 60 s, with a final elongation step at 72°C for 7 min. The amplified products were purified using an Agencourt AMPure XP Kit (Beckman Coulter, Brea, CA, USA). High-throughput sequencing was performed using Illumina MiSeq platforms (Beijing Allwegene Co., Ltd.).

### High−throughput sequencing data analysis

When adaptors and primer sequences were removed, QIIME (v1.8.0) was used to assemble the raw sequences of each sample according to the unique barcode. The Uparse algorithm of Vsearch (v2.7.1) software was used to cluster high-quality reads by operational taxonomic units (OTUs) at a similarity threshold of 97%. The Ribosomal Database Project (RDP) Classifier tool was used to divide the sequences into different taxonomic groups against the SILVA128 database. Alpha-diversity indices were then calculated with QIIME1 (v1.8.0). Principal coordinates analysis (PCoA) and heatmap figures were analyzed by R (v3.6.0) based on the Weighted Unifrac distance to analyze the structure and composition of the microbial community. Redundancy analysis (RDA) was used to analyze the multiply relationships between soil biochemical properties and microbial community composition at the genus level by R (v3.6.0). LEfSe (linear discriminant analysis (LDA) Effect Size) was employed to identify the biomarkers of each treatment with an LDA score ≥ 3.0 by Python (v2.7).

### Statistical analysis

Multiple comparisons were conducted by one-way analysis of variance (ANOVA) for yield, disease incidence, soil biochemical properties, and microbial relative abundance according to Duncan’s test (*p* < 0.05). Pearson correlation analysis was conducted to analyze the relationship between the soil biochemical properties. Spearman correlation analysis was conducted to analyze the relationship between the top twenty microbial communities in genus level and soil biochemical properties. SPSS v22.0 (SPSS Inc., Chicago, IL, United States) was used in all these analyses. SmartPLS v3.3.9 (Ringle et al., 2015) was used to construct partial least squares structural equation model (PLS-SEM) and explore the relationship between rotation system, yield, soil biochemical properties and microbial community.

## Results

### Potato yield and tuber black scurf incidence

**Figure 1:**
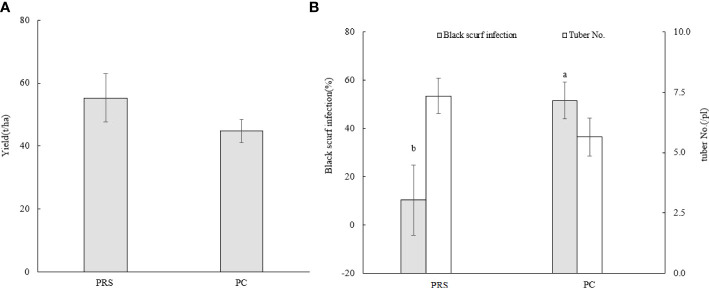
Potato yield **(A)** and black scurf infection of tubers **(B)**. PRS indicate the mean yield of potato from O treatment in 2020 and PR treatment in 2021. The grey bar indicates tuber black scurf infection and the white bar indicates tuber No. in **(B)**. Different lowercase letters at the top of the bar indicate significant differences at *P < 0.05*.

The two-year average potato yield from potato rotation system (PRS) and potato continuous cropping (PC, potato-potato-potato-potato) was showed in **Figure 1A**. PRS consisted of the potato yield of O treatment (oat-faba bean-potato-oat) in 2020 and PR treatment (potato-oat-faba bean-potato) in 2021. The crop rotation systems significantly affected tuber disease infection and tuber number of potato (**Figure 1B**). PRS increased potato yield by 23.5%, significantly decreased black scurf incidence by 80% (*p* < 0.05), and increased tuber number by 30.2% compared with PC.

### Soil chemical and biological properties

In terms of soil chemical properties, the crop rotation systems only had significant effects on soil AK (*p* < 0.05), as the content of soil AK in PR was significantly higher compared with B and O at pre-planting and B at harvest (*p* < 0.05) (**Table 1**). The value of other soil chemicals was similar between all treatments at both time points. Among all crops, both soil AP and AK content were significantly higher at harvest than at pre-planting. Compared with PR, PC reduced all soil nutrient content at both time points.

**Table 1:** Soil chemical properties at pre-planting and harvest in different cropping systems in 2021.

Treatment	AP(mg/kg)		AK(mg/kg)		AN(mg/kg)		OM(g/kg)		pH	
	Pre-planting	Harvest	Pre-planting	Harvest	Pre-planting	Harvest	Pre-planting	Harvest	Pre-planting	Harvest
PC	17.7 ± 2.1	101.7 ± 12.0 *	28.9 ± 3.9 a	219.3 ± 8.4 a*	64.8 ± 2.8	64.2 ± 4.1	14.4 ± 0.6	15.0 ± 1.5	8.1 ± 0.0	8.2 ± 0.1
PR	15.6 ± 4.7	105.3 ± 25.7 *	30.8 ± 2.3 a	228.0 ± 47.9 a*	72.4 ± 10.3	72.8 ± 9.9	16.4 ± 2.6	15.9 ± 1.8	8.1 ± 0.0	8.2 ± 0.0
B	13.9 ± 2.2	99.7 ± 26.1 *	17.7 ± 0.8 b	103.3 ± 20.1 b*	68.5 ± 7.6	66.6 ± 9.1	16.2 ± 1.9	16.6 ± 2.2	8.1 ± 0.0	8.1 ± 0.1
O	13.6 ± 1.8	98.3 ± 25.6*	20.2 ± 3.2 b	198.0 ± 35.3 a*	70.4 ± 6.4	67.3 ± 2.7	16.0 ± 0.3	15.4 ± 1.6	8.1 ± 0.0	8.1 ± 0.0

Lowercase letters within a column mean significant differences among treatments (p < 0.05), and * p < 0.05 means significant differences of the same treatment between the two time points. AP, available phosphorus; AK, available potassium; AN, available nitrogen. Data represent mean values ± SE (n = 3).

Among soil biological activities, only soil SUC activity was significantly affected (*p* < 0.05) by cropping systems, with that in O significantly higher compared with the other three cropping systems at harvest (**Table 2**). Compared with pre-planting, soil SUC of O increased by 57.5% at harvest (*p* < 0.05). Soil ACP in all treatments at harvest increased by a significant 30.9-45.4% from that at pre-planting (*p* < 0.05). Soil URE and ALP activities were similar among all treatments. However, all the enzyme activities in PC at harvest were lower than those in the other cropping systems. Soil chemical properties had significant correlation with enzyme activities (*p* < 0.05) (**Table 3**). AK was positively correlated with AP and ACP. OM was positively correlated with all enzymes and AN, but negatively correlated with pH, which in turn was significantly correlated with AN. AN was positively correlated with ALP, ACP and SUC. All these four enzymes were significantly correlated with each other (*p* < 0.05).

**Table 2:** Soil enzyme activities at pre-planting and harvest in different cropping systems in 2021.

Treatment	ALP (U/g)		ACP (U/g)		SUC (U/g)		URE (U/g)	
	Pre-planting	Harvest	Pre-planting	Harvest	Pre-planting	Harvest	Pre-planting	Harvest
PC	13270.3 ± 2400.3	15112.5 ± 1067.7	10180.7 ± 950.4	13966.7 ± 612.0 *	18.6 ± 4.0	23.5 ± 3.8 b	28.1 ± 3.7	26.2 ± 3.2
PR	13688.1 ± 4373.0	14979.5 ± 2468.0	12621.4 ± 2915.8	16541.9 ± 1798.1 *	26.7 ± 9.9	24.7 ± 5.4 b	30.4 ± 7.6	30.2 ± 5.7
B	12539.1 ± 3363.3	19243.1 ± 2358.2	11939.2 ± 1709.1	17358.7 ± 1741.8 *	24.3 ± 5.9	26.6 ± 3.6 b	28.1 ± 2.6	30.9 ± 5.8
O	12368.2 ± 1264.7	15055.5 ± 1558.9	13274.9 ± 192.7	17377.9 ± 1093.0 *	21.0 ± 2.5	33.1 ± 1.1 a*	29.2 ± 0.8	32.6 ± 4.9

Lowercase letters within a column mean significant differences among treatments (p < 0.05), and *p < 0.05 means significant differences of the same treatment between the two time points. ALP, alkaline phosphatase; ACP, acid phosphatase; SUC, sucrase; URE, urease. Data represent mean values ± SE (n = 3).

**Table 3:** Pearson correlations between soil chemical and biological properties.

	AP	AK	pH	OM	AN	ALP	ACP	SUC
AK	0.514*							
pH	0.386	0.343						
OM	-0.007	-0.017	-0.453*					
AN	0.165	0.175	-0.425*	0.811**				
ALP	0.13	0.289	-0.105	0.534**	0.498*			
ACP	0.306	0.484*	0.024	0.551**	0.531**	0.750**		
SUC	0.181	0.164	-0.07	0.582**	0.546**	0.445*	0.667**	
URE	-0.089	0.148	-0.085	0.416*	0.394	0.449*	0.470*	0.562**

*p < 0.05 and **p < 0.01 means significant correlations between two properties. ALP, alkaline phosphatase; ACP, acid phosphatase; SUC, sucrase; URE, urease; AP, available phosphorus; AK, available potassium; AN, available nitrogen.

### α-diversity of microbial community

The cropping system had significant effects on Shannon index of bacterial community and Observed_species and PD_whole_tree indices of fungal community at pre-planting and on all four α-diversity indices of bacterial community and Observed_species and Shannon indices of fungal community at harvest. (*p* < 0.05) (**Table 4**). Although all the α-diversity indices between PC and PR were not much different at harvest, they were significantly different from those of O and B. At pre-planting, all bacterial community diversity indices except the Shannon index were similar among different cropping systems, while the fungal community diversity indices of PC were lower compared with those of the other cropping systems. Even the indices of Observed_species and PD_wholw_tree were significantly lower (*p* < 0.05). At harvest, the bacterial community diversity indices of PC were the highest, and even significantly higher than those of B and O systems (*p* < 0.05). By contrast, the fungal community diversity indices of PC were the lowest, even significantly lower than those of B and O systems for Observed_species and Shannon indices (*p* < 0.05). In general, the α-diversity indices of bacterial and fungal communities of PC were higher and lower, respectively, compared with those of PR.

**Table 4:** α-diversity indices of soil samples in all cropping systems.

Period	Treatment	Chao1		Observed_species		PD_whole_tree		Shannon	
		Bacteria	Fungi	Bacteria	Fungi	Bacteria	Fungi	Bacteria	Fungi
Pre-planting	PC	4541.2 ± 144.7	979.9 ± 98.4	3297.5 ± 64.3	764.6 ± 32 b	286.5 ± 7.7	172.1 ± 6.7 b	10.1 ± 0 a	6.4 ± 0.2
	PR	4418.3 ± 114.1	1131.9 ± 60.1	3257.1 ± 39.6	876 ± 5.1 a	284.4 ± 4.4	199 ± 10.8 a	10.1 ± 0 a	6.4 ± 0.3
	B	4454.1 ± 109.6	1142.9 ± 18.0	3204.3 ± 56.6	899.3 ± 29.4 a	276.6 ± 4.2	197.2 ± 3.3 a	10 ± 0 b	6.5 ± 0.2
	O	4442.8 ± 47.1	1139.9 ± 42.0	3226.5 ± 23.2	858 ± 27.9 a	279.6 ± 1.0	192.3 ± 12.1 a	10.1 ± 0 a	6.5 ± 0.1
Harvest	PC	4917.6 ± 104.3 a	1024.3 ± 34.5	3550.1 ± 31.3 a	721.3 ± 46.4 c	306.5 ± 1.8 a	156.8 ± 8.6	10.3 ± 0 a	5 ± 0.4 b
	PR	4864.6 ± 83.5 a	1044 ± 9.2	3499 ± 15.4 a	739.3 ± 29 bc	301.8 ± 1.6 a	159.9 ± 8.2	10.3 ± 0 ab	5.4 ± 0.4 b
	B	4560.3 ± 131.4 b	1102.4 ± 81.7	3222.8 ± 67.5 b	831 ± 45.8 ab	281.5 ± 6.8 b	172.5 ± 6.5	10 ± 0.1 c	6.3 ± 0.2 a
	O	4390.6 ± 155.4 b	1091.2 ± 57.9	3237.1 ± 70.9 b	855.9 ± 34.7 a	280.4 ± 4.6 b	175.7 ± 11.2	10.2 ± 0 b	6.4 ± 0.1 a

Lowercase letters within a column mean significant differences among treatments (p < 0.05). Data represent mean values ± SE (n = 3).

### Microbial community structure and composition

**Figure 2:**
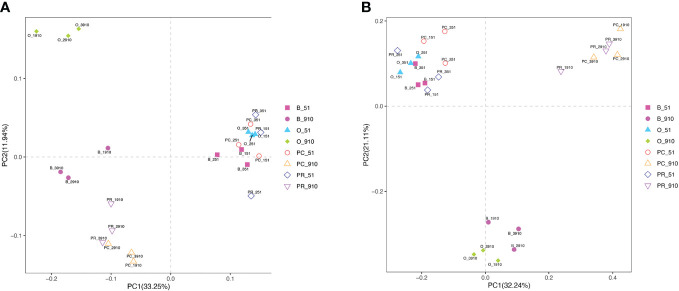
Principal coordinate analysis (PCoA) of soil bacterial **(A)** and fungal **(B)** communities. B_51, O_51, PC_51 and PR_51 indicate the samples from B, O, PC and PR treatments at pre-planting, respectively; B_910, O_910, PC_910 and PR_910 indicate the samples from B, O, PC and PR treatments at harvest.

**Figure 3:**
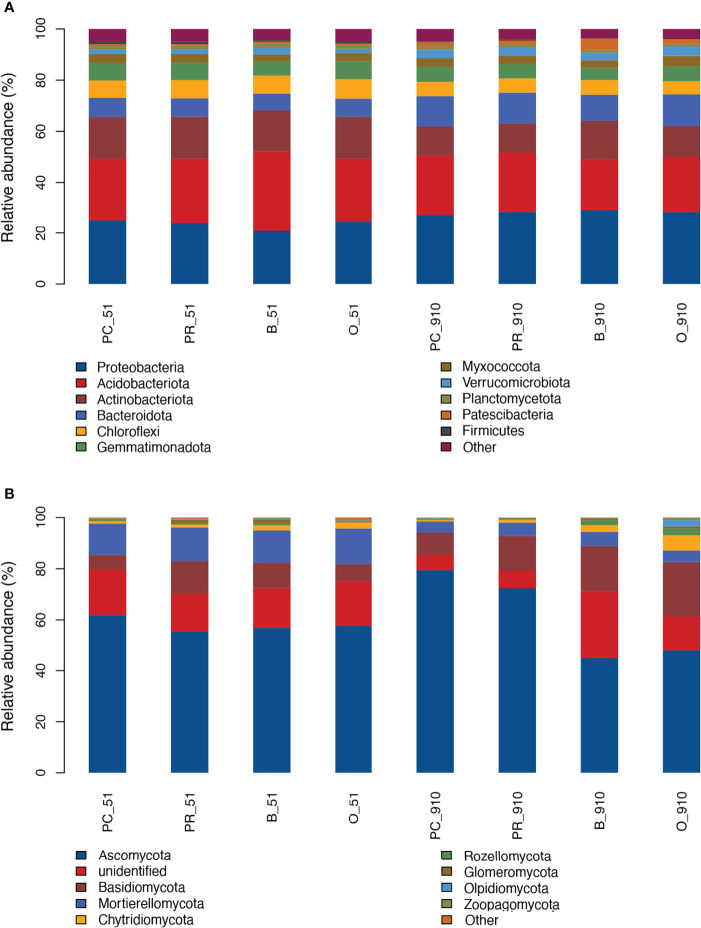
Bacterial **(A)** and fungal **(B)** community structure at the phylum level. B_51, O_51, PC_51 and PR_51 indicate the samples from B, O, PC and PR treatments at pre-planting, respectively; B_910, O_910, PC_910 and PR_910 indicate the samples from B, O, PC and PR treatments at harvest.

The different cropping systems significantly affected bacterial and fungal community structure as evident from the PCoA analysis (*p* < 0.05) (**Figure 2**). The four systems were clustered together at pre-planting and significantly different from harvest along the PCo1 axis. In terms of bacterial community, the relative abundance of 11 phyla, including Proteobacteria, Acidobacteriota, Actinobacteriota, Bacteroidetes, Gemmatimonadota, Chloroflexi, Myxococcota, Verrucomicrobiota, Planctomycetota, Firmicutes, and Patescibacteria, was more than 1% (**Figure 3A**). However, the dominant phyla with the highest relative abundance in each cropping system was not the same at pre-planting. For example, Acidobacteriota showed the highest relative abundance in B and PR, while Proteobacteria showed the highest relative abundance in O and PC. Only the relative abundance of Chloroflexi in O was significantly higher than in other cropping systems (*p <* 0.05) (**Supplementary Figure 1A**). At harvest, the relative abundance of three phyla exhibited significant differences among cropping systems (*p* < 0.05). Myxococcota in O and Patescibacteria in B had significantly higher relative abundance than in the other treatments. The relative abundance of Planctomycetota in PC was significantly higher than in B and O but similar to that in PR. Analysis of the top 20 most abundant bacterial genera revealed that 2 and 10 genera were significantly different among cropping systems at pre-planting and harvest, respectively (**Supplementary Figure 1B**). At pre-planting, the relative abundance of metagenome genus was significantly higher in PR than in the other cropping systems, and uncultured genera was significantly higher in B than in the other cropping systems. At harvest, the relative abundance of uncultured_bacterium in PC was significantly higher than in the other three systems. The relative abundance of unidentified genera was higher in B than in the other cropping systems and significantly higher than in O. The relative abundance of *Metagenome* and *Devosia* was significantly higher in O. *Allorhizobium-Neorhizobium-Pararhizobium-Rhizobium*, *Aeromicrobium*, and *Lysobacter* had higher relative abundance in B, while *Pseudomonas* and *Methylotenera* showed higher relative abundance in PR.

Among the fungal community, the relative abundance of 9 phyla, including Ascomycota, unidentified, Mortierellomycota, Basidiomycota, Chytridiomycota, Glomeromycota, Rozellomycota, Olpidiomycota, and Zoopagomycota, was more than 1% (**Figure 3B**). Five of them showed significant differences among all cropping systems at harvest (*p* < 0.05) (**Supplementary Figure 2A**). For example, the relative abundance of Ascomycota was significantly higher in PC than in the other cropping systems, and significantly higher in PR than in O and B. The relative abundance of Chytridiomycota in PC was significantly lower than in the other treatments. The relative abundance of Basidiomycota in PC and PR were similar but significantly higher than in O and B. At pre-planting, the four cropping systems were similar in all of the phyla. Eighteen of the 20 most abundant fungal genera at harvest belonged to the phyla Ascomycota and Basidiomycota, including nine genera that exhibited significant differences among all cropping systems (*p < 0.05*) (**Supplementary Figure 2B**). For example, the relative abundance of *Papiliotrema*, *Paraphoma*, *Cladophialophora*, *Stachybotrys* and *Humicola* showed the highest value in B, and even were significantly higher than in the other cropping systems except *Humicola*. The relative abundance of *Plectosphaerella* and *Colletotrichum* in PC and PR were similar, but both were significantly higher than in B and O, while *Vishniacozyma* in PC and PR showed the opposite trend. The relative abundance of *Fusarium* and *Verticillium* were higher in PC at harvest compared with other cropping systems but insignificantly (data were not shown).

**Figure 4:**
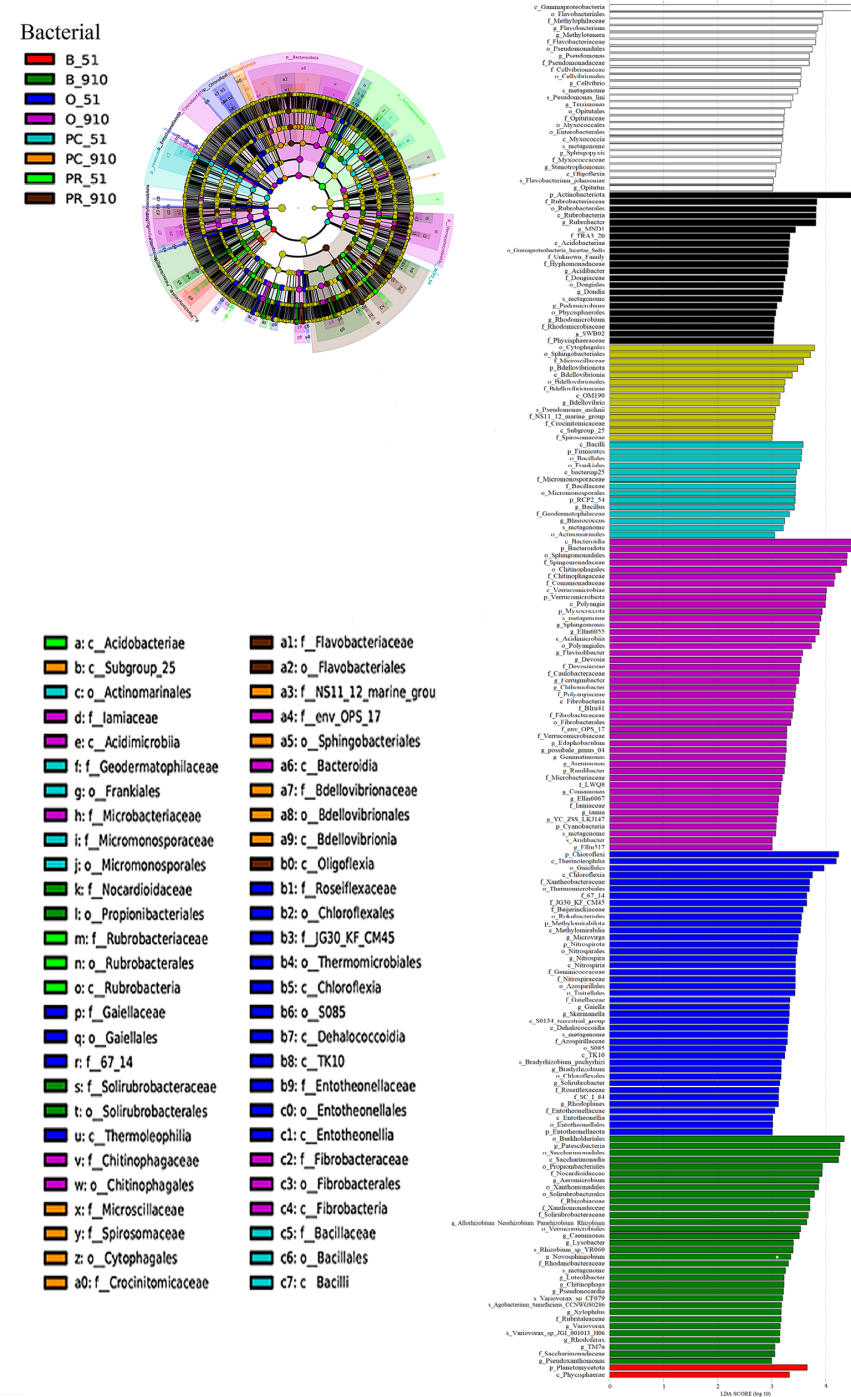
LEfSe analysis of soil bacterial community structure at different taxonomic levels among different rotation systems (LDA ≥ 3.0). The left figure is the cladogram of microbial communities. The right graph is the significant different taxa among treatments with LDA ≥3. B_51, O_51, PC_51 and PR_51 indicate the samples from B, O, PC and PR treatments at pre-planting, respectively; B_910, O_910, PC_910 and PR_910 indicate the samples from B, O, PC and PR treatments at harvest.

**Figure 5:**
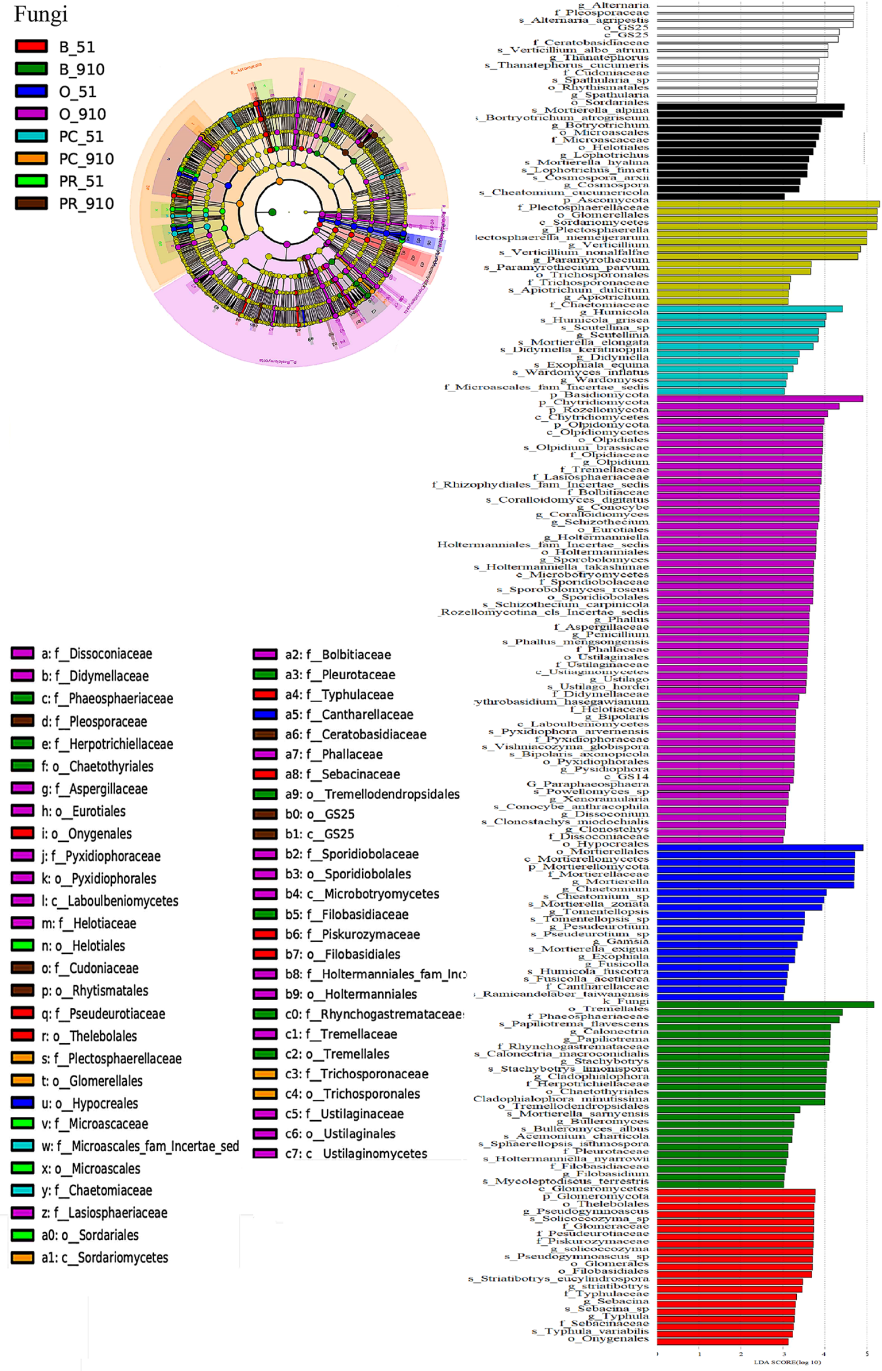
LEfSe analysis of soil fungal community structure at different taxonomic levels among different rotation systems (LDA ≥ 3.0). The left figure is the cladogram of microbial communities. The right graph is the significant different taxa among treatments with LDA ≥3. B_51, O_51, PC_51 and PR_51 indicate the samples from B, O, PC and PR treatments at pre-planting, respectively; B_910, O_910, PC_910 and PR_910 indicate the samples from B, O, PC and PR treatments at harvest.

Biomarkers of all cropping systems were identified by LEfSe, and there were 77 and 121 bacterial communities and 67 and 112 fungal communities (LDA > 3) at all taxonomic levels at pre-planting and harvest, respectively (**Figures 4**, **5**). At harvest, there were more biomarkers for each cropping system than at pre-planting. The PC treatment showed the same number of fungal biomarkers (14) and five less bacterial biomarkers (22) compared to PR treatment. At harvest, the most abundant bacterial biomarkers in PC included Cytophagales (order), Sphingobacteriales (order), Microscillaceae (family), and Bdellovibrionia (phylum), while the most abundant fungal biomarkers in PC were Ascomycota (phylum), Plectosphaerellaceae (family), Glomerellales (order), Sordariomycetes (class), *Plectosphaerella* (genus), *Plectosphaerella niemeijerarum* (species), and *Verticillium* (genus).

### Relationship Between Environmental Factors, Microbial Community and Rotation System

**Figure 6:**
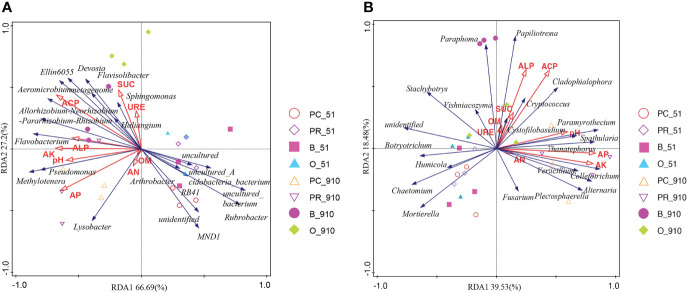
Redundancy analysis (RDA) of bacterial **(A)** and fungal **(B)** communities (genera) and soil biochemical properties. B_51, O_51, PC_51 and PR_51 indicate the samples from B, O, PC and PR treatments at pre-planting, respectively; B_910, O_910, PC_910 and PR_910 indicate the samples from B, O, PC and PR treatments at harvest.

**Figure 7:**
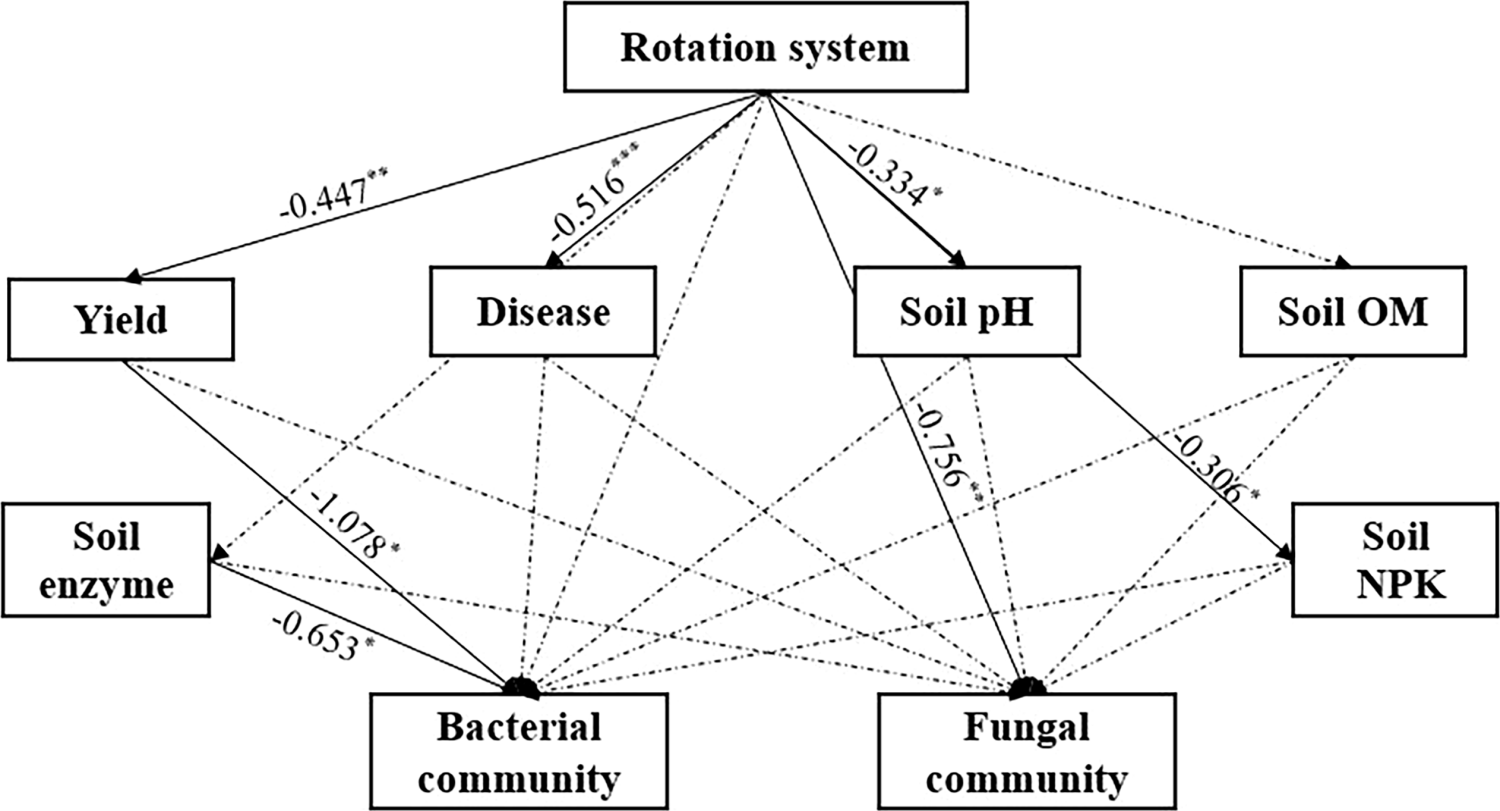
Partial least squares structural equation model (PLS-SEM) graph. Solid line and Dash line with arrow indicate significant and insignificant effect between the two measurements, respectively. Bacterial community and fungal community consisted of the top 20 bacterial and fungal genera, respectively. The numbers next to the line indicate the path coefficient. *P < 0.05, **P < 0.01 and ***P < 0.001 indicate significant effect between the two measurements.

Redundancy analysis (RDA) consisted of the soil chemicals and dominant microbial communities at the genus level (**Figure 6**). The bacterial community structure was significantly affected by soil AK, ACP, pH, SUC and AP, and the fungal community structure was significantly affected by soil AK, ACP, pH, SUC, AP and AN (*p < 0.05*). Spearman correlation analysis between environmental factors and the top 20 microbes at the genus level showed similar results to RDA analysis but differed slightly in that, soil AN had no effect on the fungal genera. In addition, most microbial communities had strong relationship with each other (**Supplementary Figure 3**). Partial least squares structural equation model (PLS-SEM) was used to assess the relationship between rotation system, environmental factors and microbial community (**Figure 7** and **Supplementary Table 1**). The results showed that rotation system had direct significant negative effects on yield, disease, soil pH and soil NPK with the path coefficients of -0.447, -0.516, -0.334 and -0.306, respectively (*p < 0.05*) and significant negative effect on fungal community with the total path coefficient of -0.756. Both soil enzyme and crop yield had direct significant negative effect on bacterial community with the path coefficients of -0.653 and -1.087, respectively (*p < 0.05*).

## Discussion

Many studies have shown that the continuous cropping system not only decreases crop yield but also increases the incidence of disease (Wright et al., 2016). Liu et al. (2014) and Qin et al. (2017) reported that potato yield decreased significantly after two years of continuous potato cropping, and by 85% after 4 years of continuous cropping (Qin et al., 2017). In the current study, potato yield decreased by an insignificant 23.5% after 4 years continuous cropping (PC) relative to potato rotation system, possibly as a result of soil texture and fertility or irrigation management. However, the incidence of potato tuber black scurf was significantly higher in PC, which was congruent with results from Bernard et al. (2014) and Larkin et al. (2011) that different rotation systems could reduce the incidence of black scurf by18 to 58% relative to PC. Interestingly, PC significantly decreased the tuber number relative to potato rotation system, which was inconsistent with the findings of Mohr et al. (2011) that crop rotations affected the number of large or small tubers but not the total number of tubers.

Different cropping systems had significant effects on soil microbial communities (Larkin et al., 2021). In this study, the rotation system had significant negative effect on fungal community. PR and PC, especially PC, increased the diversity of soil bacterial community and reduced the diversity of soil fungal community, which was consistent with the results of cotton (Xi et al., 2021) and lily (Shi et al., 2021) cropping systems and a 2-11 years continuous potato cropping systems (Zhao et al., 2020). However, this finding was opposite to a study on a 7-year continuous potato cropping system, which showed that the Shannon and Chao1 indices of the fungal community remained consistent over the years but the bacterial community significantly decreased (Liu et al., 2014; Qin et al., 2015); this might be caused by soil type, and was supported by the study of İnceoğlu et al. (2012) that bacterial communities from the rhizosphere of the same potato cultivar were quite diverse in different soil.

The cropping rotation system significantly affected microbial community structure and composition (*p* < 0.05). In terms of bacterial community, the predominant phyla at pre-planting were similar to harvest. Chloroflexi, which can degrade organic residues (Zeng et al., 2021), was the only phylum that exhibited significant differences in relative abundance among treatments and was significantly higher in O followed by B, PR, and PC at pre-planting. The possible reason was that in all the treatments except for PC, oats were planted in one of the four years and oat straw returned to the field, the degradation of which may lead to higher abundance of Chloroflexi. At harvest, Myxococcota, Patescibacteria, and Planctomycetota showed significant differences in relative abundance among treatments. The abundance of Myxococcota was improved by application of organic materials (Zhang et al., 2022), which was higher in a yam rotation cropping system compared with in continuous cropping systems (Zhou et al., 2021). Patescibacteria is involved in nitrogen, sulfur, and iron cycling (Herrmann et al., 2019). In the present study, the relative abundance of Myxococcota in O and that of Patescibacteria in B were both significantly higher compared with those in other cropping systems. In pepper rotation and intercropping systems, the abundance of Planctomycetota and Acidobacteria increased significantly relative to a continuous cropping system, which accounted for the reduction in soil-borne disease (Dong et al., 2019). However, in the current study, the relative abundance of Planctomycetota was higher in PR and PC relative to B and O. In the present study, Proteobacteria was the dominant phylum in all treatments, which was consistent with the studies that Proteobacteria was, reported as the predominant phylum in potato cultivated soil (Liu et al., 2014; Zhao et al., 2020). At harvest, six known genera of the top 20 bacterial genera presented significant differences among different cropping systems, five of which belonged to the phylum Proteobacteria, including *Pseudomonas*, *Devosia*, *Allorhizobium-Neorhizobium-Pararhizobium-Rhizobium*, *Lysobacter*, and *Methylotenera*. Members of the genus *Devosia* are involved in nitrogen-fixing (Rivas et al., 2002) and straw degradation together with *Allorhizobium-Neorhizobium-Pararhizobium-Rhizobium* (Wang et al., 2019). *Aeromicrobium* can produce erythromycin and may inhibit the Gram-negative bacteria in the soil (Reeves et al., 2004). The abundance of *Devosia* in O and *Allorhizobium-Neorhizobium-Pararhizobium-Rhizobium* and *Aeromicrobium* in B was significantly higher than in other cropping systems. Fluorescent *Pseudomonas* suppressed soil-borne disease (Farah et al., 2008) and *Pseudomonas* sp. RU47 improved P uptake and plant growth (Nassal et al., 2018). However, *Pseudomonas palleroniana* caused potato tuber soft rot (Peng et al., 2022). In the current study, *Pseudomonas* showed significantly higher relative abundance in PC and PR than in B and O, and the functions of members of this genus in these crop systems need to be studied further.

For the fungal community, the main phyla at pre-planting were also similar to harvest. All these fungal phyla showed similar changes among cropping systems at pre-planting, but the first four showed significant differences at harvest. Three of them including Ascomycota, Basidiomycota and Chytridiomycota predominantly belong to the saprotrophic soil fungi and are involved in aerobic cellulose degradation (de Boer et al., 2005), which may improve soil fertility as well as increasing disease incidence during the decomposition and decay process. The relative abundance of the phylum Ascomycota was significantly higher in PC compared with that in PR followed by B and O, but the relative abundance of the phylum Basidiomycota was significantly lower in PC than in O and B. At harvest, eight known genera of the top 20 fungal genera presented significant differences among cropping systems. Members of the genus *Colletotrichum* (Hyde et al., 2009), *Plectosphaerella* (Carlucci et al., 2012; Li et al., 2017) and *Paraphoma* (Cao et al., 2020) are harmful pathogens, and *Paraphoma* can degrade plant residues and biodegradable plastic (Sameshima-Yamashita et al., 2016). The genera *Colletotrichum* and *Plectosphaerella* belonging to phylum Ascomycota presented significantly higher relative abundance in PC and PR than in O and B, which could increase the disease incidence especially in PC. The relative abundance of the genus *Paraphoma* was significantly higher in B than in other treatments, which may be related to plant residue degradation. Genera *Stachybotrys*, *Cladophialophora*, *Humicola* and *Papiliotrema* showed the highest abundance in B relative to in other cropping systems. Members of the genus *Papiliotrema* can control head blight (Khan et al., 2001) and crown rot (Liu et al., 2021) of wheat caused by *Fusarium*, which also causes potato dry rot and wilt. The incidence of the two diseases increased with increasing years of continuous cropping (Mendes et al., 2013). *Stachybotrys elegans* can decrease *Rhizoctonia solani* infection due to degradation of the host hyphae and cell wall of sclerotia (Benyagoub et al., 1994). The abundance of *Rhizoctonia solani*, which causes black scurf, was significantly higher in PC relative to that in PR. The genus *Cladophialophora* includes many plant saprophytes and endophytes, which can degrade cell walls of dead host plants or aromatic-ring based compounds (Davey and Currah, 2007).

Soil nutrient imbalances is one of the main obstacles of continuous cropping (Puniya et al., 2019). In the present study, PC treatment reduced all soil nutrient content and enzyme activities compared to the other three cropping systems, with its value close to that of PR. This was similar to the results from Ma et al. (2015) that soil nutrients did not show significant deficiency and imbalance between PC and PR but the structure of soil microbial community changed significantly. Soil enzymes are predominantly produced by soil microbes and plant roots (Schloter et al., 2003) and play a key role in nutrient cycling and energy flow in the soil ecosystem (Wang et al., 2016). In the current study, soil enzyme had direct significant effect on bacterial community. Li et al. (2018) reported that soil SUC activity was significantly higher in oat rotation and continuous cropping than in potato continuous cropping, which lead to the degradation of plant residues and an increase in soil organic matter. Similar results were obtained in the current study that SUC activity and phyla Chloroflexi related to organic residues degradation were significantly higher in O. Soil physical and chemical properties and enzymes can affect microbial community abundance and composition (Qin et al., 2017). In this study, soil AK, ACP, SUC, pH and AP affected both bacterial and fungal community significantly, which was in agreement with that soil fungal community was strongly influenced by soil AP (Li et al., 2020) and pH (Zhao et al., 2014). In addition, the rotation system had significant negative effect on fungal community, which was in consistent with the results from Wang et al. (2022) that fungal community was more sensitive to the rotation system.

At pre-planting, the bacterial and fungal community diversity and structure as well as soil chemical properties were similar among all four cropping systems, which could be mainly caused by the low temperature in winter (average temperature -12.5°C) that kept the relative abundance of soil microorganisms in a low level (Zhang et al., 2017) or caused the death of some sensitive organisms. (Shi et al., 2015). Additionally, the microbial community and soil chemical properties changed significantly at harvest than at pre-planting. So, further studies need to determine if the crop exudates recruited some specific microorganisms or they had strong mutual relations to change the diversity and structure of soil microorganisms leading to yield reduction and disease aggravation.

## Conclusion

Growing oat or faba bean can increase microorganisms related to plant residues degradation, nitrogen cycling and disease suppression. At pre-planting, the soil biochemical properties and microbial community among four different cropping systems were similar after a long cold winter. However, at harvest, a 4-year potato continuous cropping system (PC) reduced potato yield and tuber number and significantly increased the incidence of black scurf relative to PR. Furthermore, PC also decreased fungal community diversity, soil nutrient contents, and soil enzyme activities, and increased bacterial community diversity compared with the other treatments. Soil AK, ACP, SUC, pH and AP were the key factors to affect bacterial and fungal community structure. In summary, oat and faba bean can increase soil beneficial microorganisms and maintained a healthy ecosystem, thus potato rotated with them can decrease the incidence of tuber black scurf and increase potato yield.

## Data availability statement

The datasets presented in this study can be found in onlinerepositories (https://www.ncbi.nlm.nih.gov/bioproject/PRJNA741081/).

## Author contributions

JQ, JL and GL designed the research. JL and GL supervised the whole work, JQ carried out the experiments, collected and analyzed the data, and prepared the manuscript. CB, SD and WW provided suggestions for data analysis. All authors approved the manuscript for publication.

## Funding

This research was funded by National Key R&D Program of China (2020YFD1000800) and China Agriculture Research System (CARS-09-P11).

## Acknowledgments

We thank Xiuhan Liang for language revision and Shuxin Li for figures modification.

## Conflict of interest

The authors declare that the research was conducted in the absence of any commercial or financial relationships that could be construed as a potential conflict of interest.

The reviewer XC declared a shared second affiliation with the authors at the time of the review.

## Publisher’s note

All claims expressed in this article are solely those of the authors and do not necessarily represent those of their affiliated organizations, or those of the publisher, the editors and the reviewers. Any product that may be evaluated in this article, or claim that may be made by its manufacturer, is not guaranteed or endorsed by the publisher.

## Supplementary material

Supplementary Figure 1 Relative abundance of dominant bacterial phyla (A) and genera (B) among different treatments. Lowercase letters indicate significant differences at *P* < *0.05* level. B_51, O_51, PC_51 and PR_51 indicate the samples from B, O, PC and PR treatments at pre-planting, respectively; B_910, O_910, PC_910 and PR_910 indicate the samples from B, O, PC and PR treatments at harvest.

Supplementary Figure 2 Relative abundance of dominant fungal phyla (A) and genera (B) among different treatments. Lowercase letters indicate significant differences at *P* < *0.05* level. B_910, O_910, PC_910 and PR_910 indicate the samples from B, O, PC and PR treatments at harvest.

Supplementary Figure 3 Correlation analysis of soil biochemical properties and the dominant bacterial (A) and fungal (B) genera. * and ** indicate significant differences between two indices at *P* < *0.05* and *P* < *0.01* level, respectively. ALP, alkaline phosphatase; ACP, acid phosphatase; SUC, sucrase; URE, urease; AP, available phosphorus; AK, available potassium; AN, available nitrogen.
