# Supplementary material for: Effects of different rotation cropping systems on potato yield, rhizosphere microbial community and soil biochemical properties
Source: Front Plant Sci. 2022 Sep 29;13:999730. doi: 10.3389/fpls.2022.999730 (PMC9559605; doi:10.3389/fpls.2022.999730)
Supplement: Supplementary file 1 [file Table_1.docx]

**Supplementary material**

**Supplementary Table 1** The path coefficient evaluation of direct and indirect effects among all the measurements by PLS-SEM. Bacterial community and fungal community consisted of the top 20 bacterial and fungal genera, respectively

| Indictors | Direct  effect | Indirect effect | Total effect | Direct effect analysis | | Total effect analysis | |
| --- | --- | --- | --- | --- | --- | --- | --- |
|  |  |  |  | T Statistics | P value | T Statistics | P value |
| Disease->Bacterial community | -0.064 | 0 | -0.064 | 0.286 | 0.778 | 0.286 | 0.778 |
| Disease->Fungal community | 0.059 | 0 | 0.059 | 0.184 | 0.856 | 0.184 | 0.856 |
| Rotation system->Bacterial community | 0.114 | 0.248 | 0.362 | 0.539 | 0.105 | 1.690 | 0.105 |
| Rotation system->Disease | -0.516 | 0 | -0.516 | 4.126 | 0.000 | 4.126 | 0.000 |
| Rotation system->Fungal community | -0.524 | -0.232 | -0.756 | 1.970 | 0.062 | 3.565 | 0.002 |
| Rotation system->Soil NPK | -0.306 | 0 | -0.306 | 2.325 | 0.030 | 2.325 | 0.030 |
| Rotation system->Soil OM | 0.200 | 0 | 0.200 | 1.106 | 0.281 | 1.106 | 0.281 |
| Rotation system->Soil enzyme | 0.293 | 0 | 0.293 | 1.425 | 0.168 | 1.425 | 0.168 |
| Rotation system->Soil pH | -0.334 | 0 | -0.334 | 2.171 | 0.041 | 2.171 | 0.041 |
| Rotation system->Yield | -0.447 | 0 | -0.447 | 3.394 | 0.003 | 3.394 | 0.003 |
| Soil NPK->Bacterial community | 0.421 | 0 | 0.421 | 1.223 | 0.234 | 1.223 | 0.234 |
| Soil NPK->Fungal community | -0.695 | 0 | -0.695 | 1.001 | 0.328 | 1.001 | 0.328 |
| Soil OM->Bacterial community | 0.339 | 0 | 0.339 | 0.943 | 0.356 | 0.943 | 0.356 |
| Soil OM->Fungal community | -0.706 | 0 | -0.706 | 1.391 | 0.178 | 1.391 | 0.178 |
| Soil enzyme->Bacterial community | -0.653 | 0 | -0.653 | 2.348 | 0.028 | 2.348 | 0.028 |
| Soil enzyme->Fungal community | 0.554 | 0 | 0.554 | 1.270 | 0.217 | 1.270 | 0.217 |
| Soil pH->Bacterial community | 0.042 | 0 | 0.042 | 0.130 | 0.898 | 0.130 | 0.898 |
| Soil pH->Fungal community | -0.397 | 0 | -0.397 | 0.797 | 0.434 | 0.797 | 0.434 |
| Yield->Bacterial community | -1.078 | 0 | -1.078 | 2.364 | 0.027 | 2.364 | 0.027 |
| Yield->Fungal community | 1.269 | 0 | 1.269 | 1.805 | 0.085 | 1.805 | 0.085 |

**Supplementary Figure 1|** Relative abundance of dominant bacterial phyla (A) and genera (B) among different treatments. Lowercase letters indicate significant differences at *P* < *0.05* level. B_51, O_51, PC_51 and PR_51 indicate the samples from B, O, PC and PR treatments at pre-planting, respectively; B_910, O_910, PC_910 and PR_910 indicate the samples from B, O, PC and PR treatments at harvest.

**Supplementary Figure 2|** Relative abundance of dominant fungal phyla (A) and genera (B) among different treatments. Lowercase letters indicate significant differences at *P* < *0.05* level. B_910, O_910, PC_910 and PR_910 indicate the samples from B, O, PC and PR treatments at harvest.

**Supplementary Figure 3|** Correlation analysis of soil biochemical properties and the dominant bacterial (A) and fungal (B) genera. * and ** indicate significant differences between two indices at *P* < *0.05* and *P* < *0.01* level, respectively. Abbreviations: ALP, alkaline phosphatase; ACP, acid phosphatase; SUC, sucrase; URE, urease; AP, available phosphorus; AK, available potassium; AN, available nitrogen.
