# Supplementary figures and images for: Effects of different rotation cropping systems on potato yield, rhizosphere microbial community and soil biochemical properties
Source: Front Plant Sci. 2022 Sep 29;13:999730. doi: 10.3389/fpls.2022.999730 (PMC9559605; doi:10.3389/fpls.2022.999730)

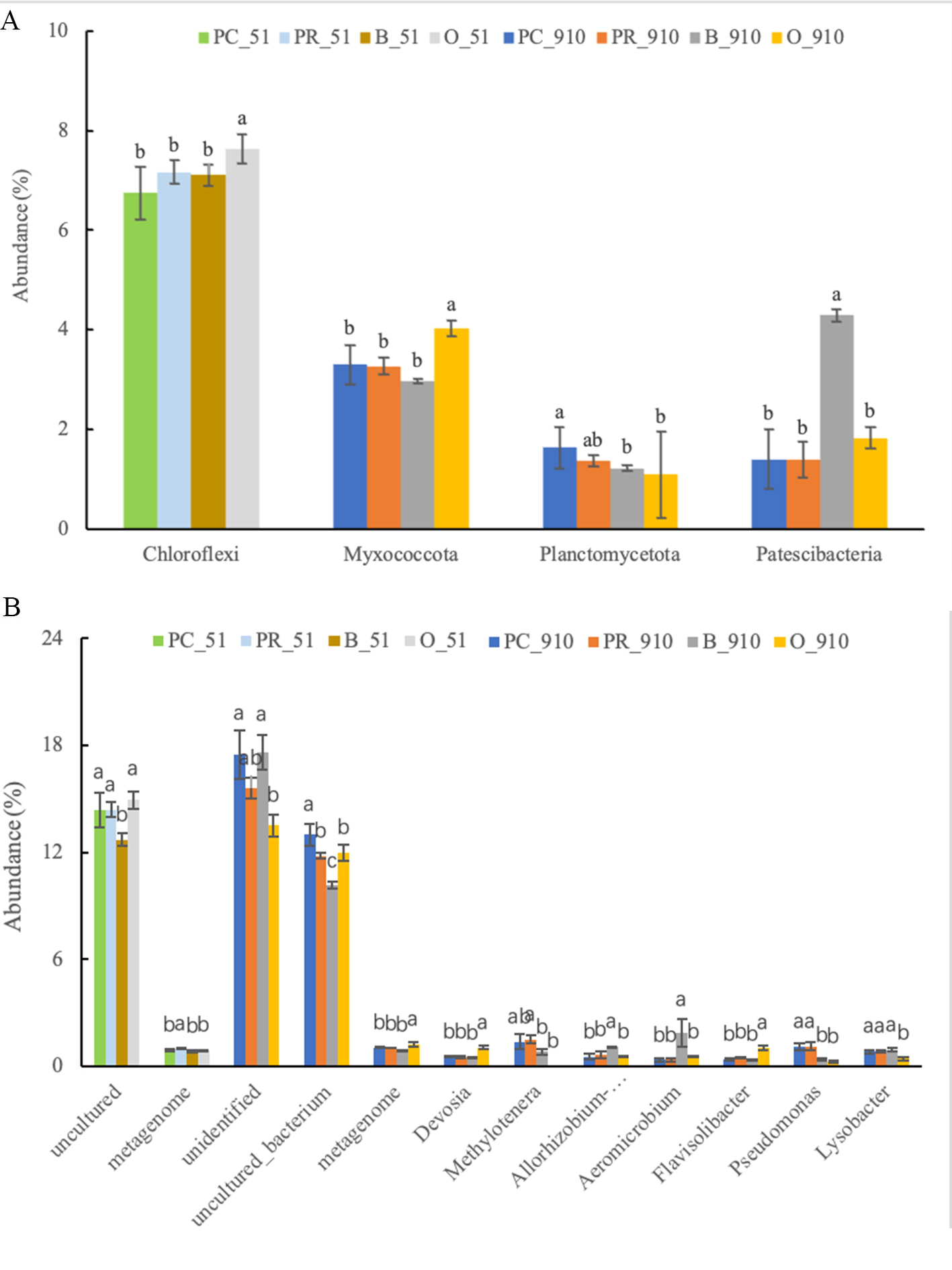

Supplement: Supplementary Figure 1 — Relative abundance of dominant bacterial phyla (A) and genera (B) among different treatments. Lowercase letters indicate significant differences at P < 0.05 level. B_51, O_51, PC_51 and PR_51 indicate the samples from B, O, PC and PR treatments at pre-planting, respectively; B_910, O_910, PC_910 and PR_910 indicate the samples from B, O, PC and PR treatments at harvest. [file Image_1.tif]

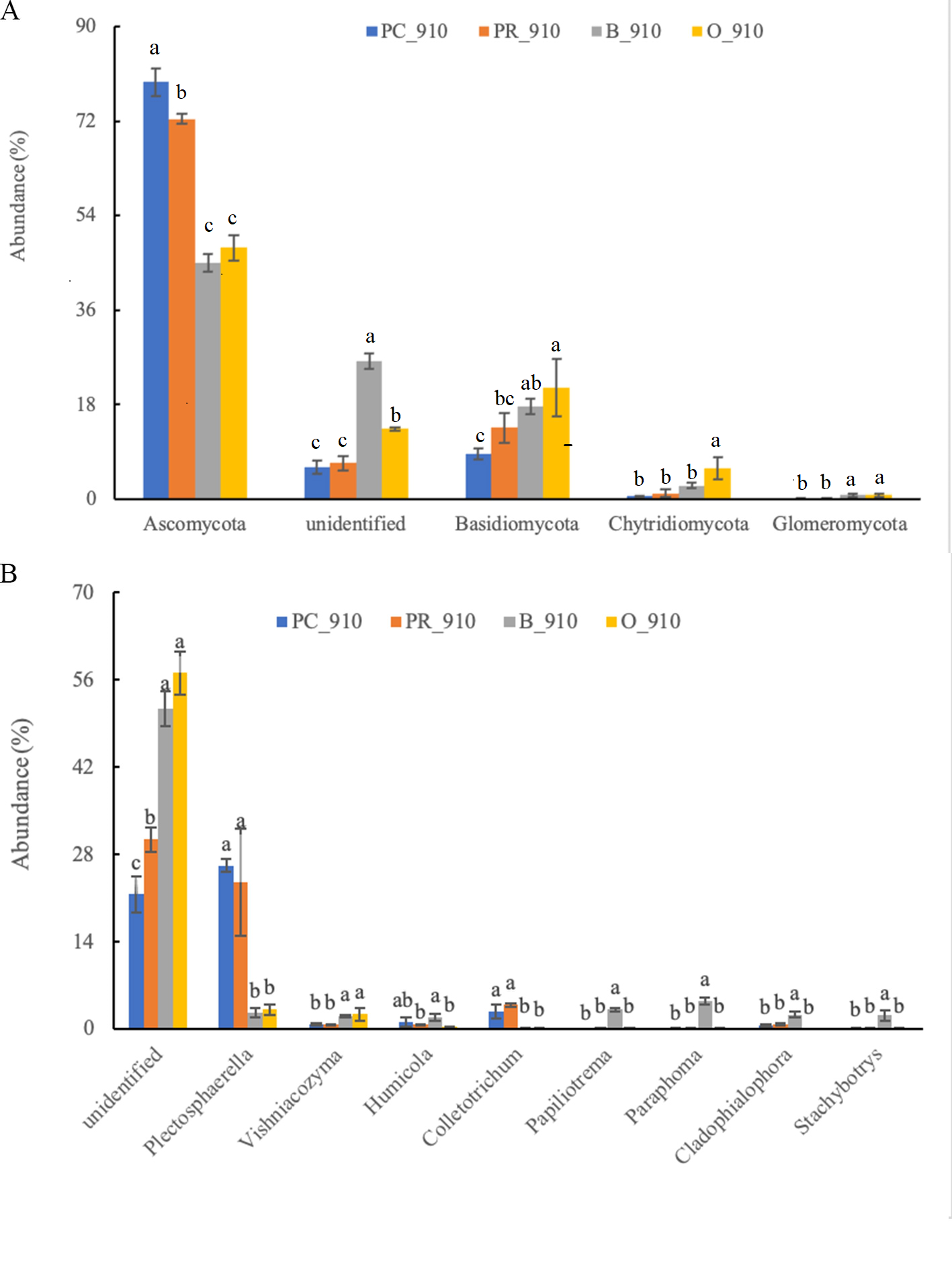

Supplement: Supplementary Figure 2 — Relative abundance of dominant fungal phyla (A) and genera (B) among different treatments. Lowercase letters indicate significant differences at P < 0.05 level. B_910, O_910, PC_910 and PR_910 indicate the samples from B, O, PC and PR treatments at harvest. [file Image_2.tif]

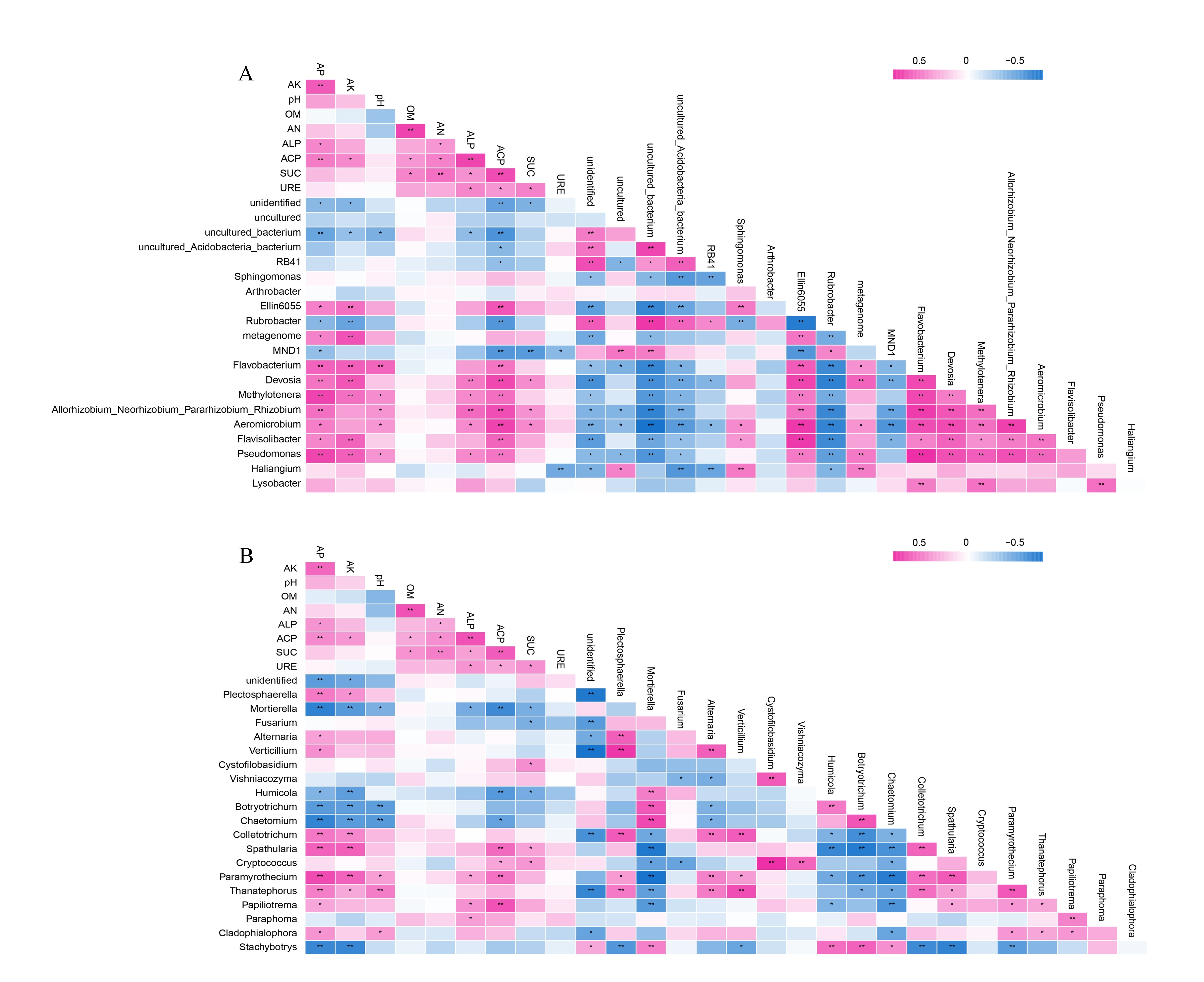

Supplement: Supplementary Figure 3 — Correlation analysis of soil biochemical properties and the dominant bacterial (A) and fungal (B) genera. * and ** indicate significant differences between two indices at P < 0.05 and P < 0.01 level, respectively. ALP, alkaline phosphatase; ACP, acid phosphatase; SUC, sucrase; URE, urease; AP, available phosphorus; AK, available potassium; AN, available nitrogen. [file Image_3.tiff]
